# Supplementary material for: Batch Distillation Data for Developing Machine Learning Anomaly Detection Methods
Source: Sci Data. 2026 Mar 31;13:513. doi: 10.1038/s41597-026-07124-3 (PMC13044305; doi:10.1038/s41597-026-07124-3)
Supplement: Supplementary file 1 — Supplementary Information [file 41597_2026_7124_MOESM1_ESM.pdf]

Supplementary Information for Batch Distillation Data for Developing Machine Learning Anomaly Detection Methods

Justus Arweiler,† Indra Jungjohann,† Aparna Muraleedharan,‡ Heike Leitte,¶ Jakob Burger,‡ Kerstin Münnemann,† Fabian Jirasek,*,† and Hans Hasse†

†*Laboratory of Engineering Thermodynamics, RPTU Kaiserslautern, Erwin-Schrödinger-Straße 44, 67663 Kaiserslautern, Germany*

‡*Technical University of Munich, Campus Straubing for Biotechnology and Sustainability, Laboratory for Chemical Process Engineering, Uferstraße 53, 94315 Straubing, Germany*

¶*Department of Computer Science, RPTU Kaiserslautern, Erwin-Schrödinger-Straße 44, 67663 Kaiserslautern, Germany*

E-mail: fabian.jirasek@rptu.de

List of Figures

S1 ^{1}H NMR spectrum of a mixture containing (ethanol + 2-propanol). 8

S2 ^{1}H NMR spectrum of a mixture containing (1-butanol + 2-propanol + water). 9

S3 ^{1}H NMR spectrum of a mixture containing (acetone + 1-butanol + methanol). 10

S4 Residue curves for the system (acetone + 1-butanol + methanol) at 1 bar. . 11

S5 Residue curves for the system (1-butanol + 2-propanol + water) at 1 bar. . . 12

S6 Availability of data modes in the dataset. 13

List of Tables

S1 Mapping of the labels in the P&I flowsheet (separate file)

S2 Suppliers of main components of the plant 3

S3 Purities and manufacturers of chemicals 11

S4 Interaction parameters τ_{ij} of the NRTL model. 12

S5 Operating points of experiments in the dataset and number of anomalies observed for each operating point (seperate file)

Additional Information on the Batch Distillation Plant

Overview

The mapping of the labels in the P&I flowsheet shown in Figure 1 of the main paper to the corresponding parts of the plant is given in Supplementary Table S1. Information on the suppliers of the parts of the plant is given in Table S2.

Table S2: Suppliers of main components of the plant. Product labels are reported as available.

Label	Supplier	Product label
AV201, AV205	Rheodyne (IDEX Health & Science)	MX, 2-position 6-port
AV708	Bürkert Fluid Control Systems	Typ 2821
AV709		Typ 6013
AV716	Gemü	Typ 102
C001, C002, C003	Iludest Destillationsanlagen	LM 2/S
V001, V002, V003		
FT201	Bronkhorst	miniCoriFlow M12
FT703, FT704		miniCoriFlow M14-ABD-330-S
FYI702	Gems	Rotorflow RFO
H001	Julabo	LC4
H002	HS-Heizelemente	Heating cartridge with Pt100
H701	SAF Wärmetechnik	
H702		
H704, H706, H708		
LS701, LS702	Omron	E3H2-DS30B4M-M1
P201	FLUSYS	WADose Lite HP
P301	Vacuubrand	MD 4C NT
P701	Verder	VGS015
P702		VGS015
PDI701, PDI702	BD sensors	DMD 831
PY23	MKS	Baratron 600
QR201	Magritek	Spinsolve 80 ULTRA Carbon
TCU1	Julabo	HE-4
TCU2	Huber	TC-45

Safety Measures

To ensure the operational safety of the batch distillation process, several measures were implemented. The laboratory-scale distillation plant was placed in a fume hood and set on

a drip pan to prevent contamination of the laboratory in the event of a leak. The pressure relief valve PRV001 was filled with paraffin oil to ensure a tight seal against ambient air inflow when operating the batch distillation plant under vacuum conditions.

Several safety shut-down measures were implemented. All heating elements are shut down if either the level sensor LS701 in the reboiler vessel V001 signals "dry" for more than 10 seconds, or if the flow of cooling water FYI702 drops below a pre-defined threshold for more than 10 seconds, or if the pressure difference between the bottom and top of the columns section exceeds a pre-defined threshold. Furthermore, all heating elements shut down if the temperature of any heating element exceeds the lowest ignition point of any component in the feed mixture of the respective experiment. The plant is secured against accidental switch-on after a power loss and shuts down if connection to the process control system is lost for more than 30 seconds.

Ambient Data

Prior to each experimental run, the ambient conditions were measured (pressure, temperature, and humidity in the laboratory, and, additionally, the outside temperature). The ambient pressure was measured with a mercury barometer (Lambrecht meteo GmbH); pressure readings were corrected for altitude, temperature, capillary, and latitude according to [1]. For measuring ambient humidity and temperature, a Testo 635-1 temperature and humidity measuring instrument (Testo Ltd) was used. Humidity was measured with an accuracy of 2% of the reading, and temperature with ± 0.3 K. For the outside temperature, the value from the Deutsche Wetterdienst in Kaiserslautern was used.

Calibration of Sensors

Mass flux Sensors

The sensors FT703 and FT704, which measure the mass flow of the withdrawn product and the reflux, were calibrated by the manufacturer using a calibration-by-comparison method traceable to national standards of the Dutch Metrology Institute VSL.

Pressure Sensors

The sensor PY23, which measures the absolute pressure in the batch distillation plant, was calibrated by the manufacturer in accordance with ISO/IEC 17025 using a Fluke forced-piston gauge transfer standard, identification number 000210609, traceable to the United States National Institute of Standards and Technology (NIST). The differential pressure sensors PDI701 and PDI702 were calibrated by the manufacturer; the authors could obtain no further information.

Temperature Sensors

The temperature sensors monitoring the temperatures inside the plant, T703, T705, T709, T711, and T712, were calibrated with a reference temperature calibration using an Isotech MicroK-100 Thermometry Bridge (Isothermal Technology Limited) combined with a Rosemount 162CE Long-Stem Standard Platinum resistance thermometer (Emerson Electric Company). The temperature sensors T701, T702, T704, T706, T708, T001, and T002 embedded in the plant's heating elements were calibrated by the manufacturers. We did not receive any related documents and therefore assume the standard measurement uncertainty of the used Pt100 temperature classes.

Offline Gas Chromatography

For offline measurement of mixture compositions in the plant, samples were drawn from V001 and V002 every 15 to 25 minutes, depending on the operating point of the experiment. First, the sample line was rinsed by drawing 1 ml of sample through it. Then, at least 0.5 ml of the sample was drawn. The mass of the samples was measured and recorded. Each sample was prepared with the internal standard 1,4-dioxane at a 4:1 mass ratio and analyzed on a gas chromatograph (GC) (Agilent 7890A). For the system (acetone + 1-butanol + methanol), as well as for the system (ethanol + 2-propanol), an Agilent Rtx-Wax column with a length of 30 meters, an inner diameter of 320 µm, and a 1 µm film thickness was used with a flame ionization detector. For the analysis of the system (1-butanol + 2-propanol + water), an Agilent HP-InnoWax column with a length of 30 meters, an inner diameter of 320 µm, and 0.5 µm film thickness was used in combination with a thermal conductivity detector.

The GC analysis was calibrated using samples with known mass ratios and the same internal standard, 1,4-dioxane. For the system (1-butanol + 2-propanol + water), 10 samples, for the system (acetone + 1-butanol + methanol), 11 samples, and for the system (ethanol + 2-propanol), 9 samples were used.

Online NMR Spectroscopy

Online concentration measurements were performed using a Spinsolve 80 ULTRA Carbon benchtop NMR spectrometer (Magritek, QR201). A glass flow cell supplied by Magritek was positioned in the magnet bore; it consisted of a 1 mm inner-diameter capillary that expanded to 4 mm at the measuring coil. The spectrometer was initially shimmed with a reference sample of 95% D_2O / 5% H_2O, yielding a signal-to-noise ratio of 50250 and a 0.34 Hz linewidth at 50% height. Before each experiment, a first- and second-order "quickshim" was performed while the process mixture was circulated through the cell at 0.2 ml min^{-1}. During operation, single-scan ^{1}H NMR spectra were acquired every minute with a 90° flip

angle and an acquisition time of 3.2 s. Spectra were processed using the Python library `NMRglue`[2]. An exponential line broadening of 1 Hz and zero-filling by a factor of 8 were applied, followed by automatic baseline correction using the adaptive penalized least-squares algorithm implemented in `pybaselines`[3–5]. Voigt profiles were then fitted to individual peaks with `DEEP picker`[6], providing peak positions, areas, and confidence values. To avoid errors from overlapping peaks, only distinct, non-overlapping spectral regions were used for each mixture.

Because internal standards cannot be added during dynamic operation, the first peak in the spectrum was fixed to a reference chemical shift. The mole fraction x_i of compound i was determined from the summed areas A_i of its multiplets via

$$x_i = \frac{\frac{A_i}{N_{\mathrm{H},i}}}{\sum_{i=0}^{N_{\mathrm{Mix}}} \frac{A_i}{N_{\mathrm{H},i}}} \tag{S1}$$

where $N_{\mathrm{H},i}$ is the number of contributing protons in compound i and N_{Mix} the number of components.

NMR spectra were collected once per minute, whereas plant sensor data were logged every second; thus, missing values were linearly interpolated. As NMR measurements are limited by flow-induced line broadening[7], the sampling pump P201 operated at 0.2 ml min^{-1}, resulting in a measurable delay between sampling and spectrum acquisition. This delay $t_{\text{NMR-delay}}$ was determined from the known tubing volume and instantaneous pump rate $\dot{V}$:

$$t_{\text{NMR-delay}} = \frac{\sum \dot{V}_{\mathrm{FT201}}}{V_{\text{Sample position-QR201}}} \tag{S2}$$

The final dataset maps each spectrum to the corresponding sampling time in the distillation process. Peak assignments for the studied mixtures are shown in Figures S1-S3, together with information on the peaks that were used for the quantitative evaluation.

The NMR magnet was thermostated at 26.5 °C. Since active cooling was unavailable, online monitoring had to be suspended during periods of elevated ambient temperature.

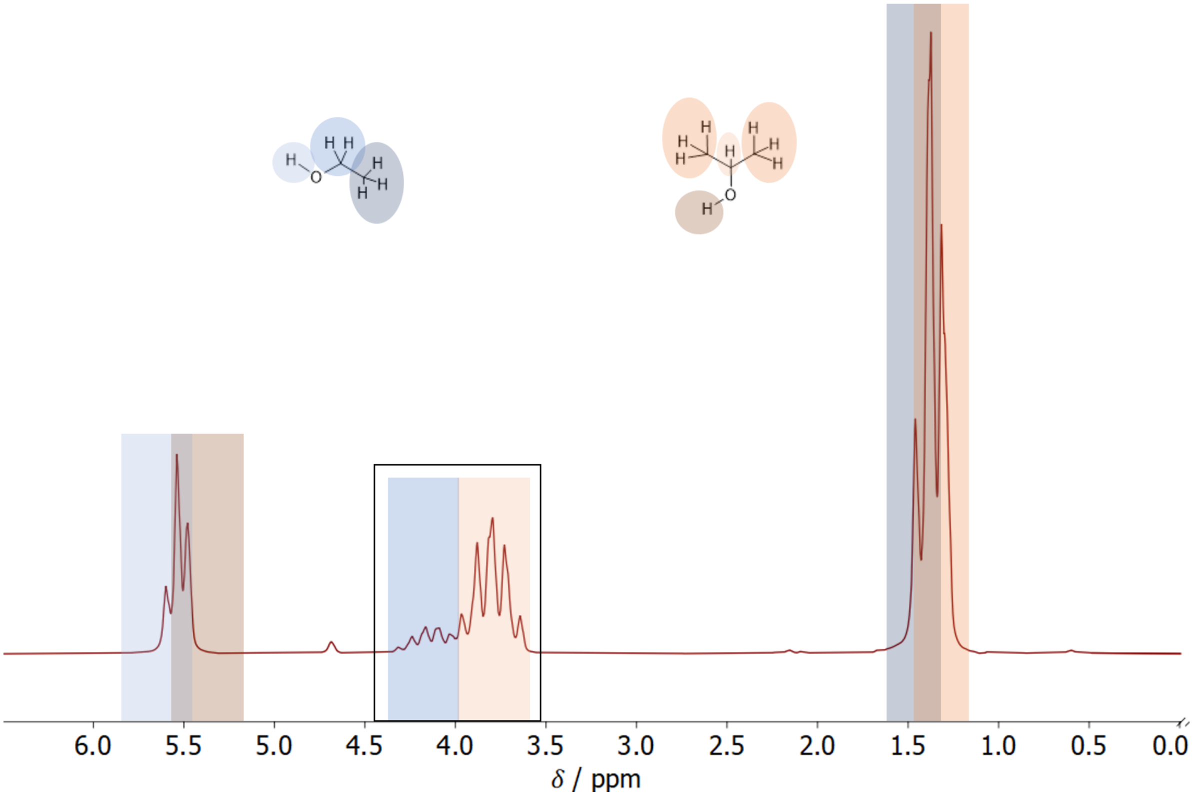

Figure S1: ^{1}H NMR spectrum of a mixture containing (ethanol + 2-propanol). The black box marks the spectral region used for quantitative analysis.

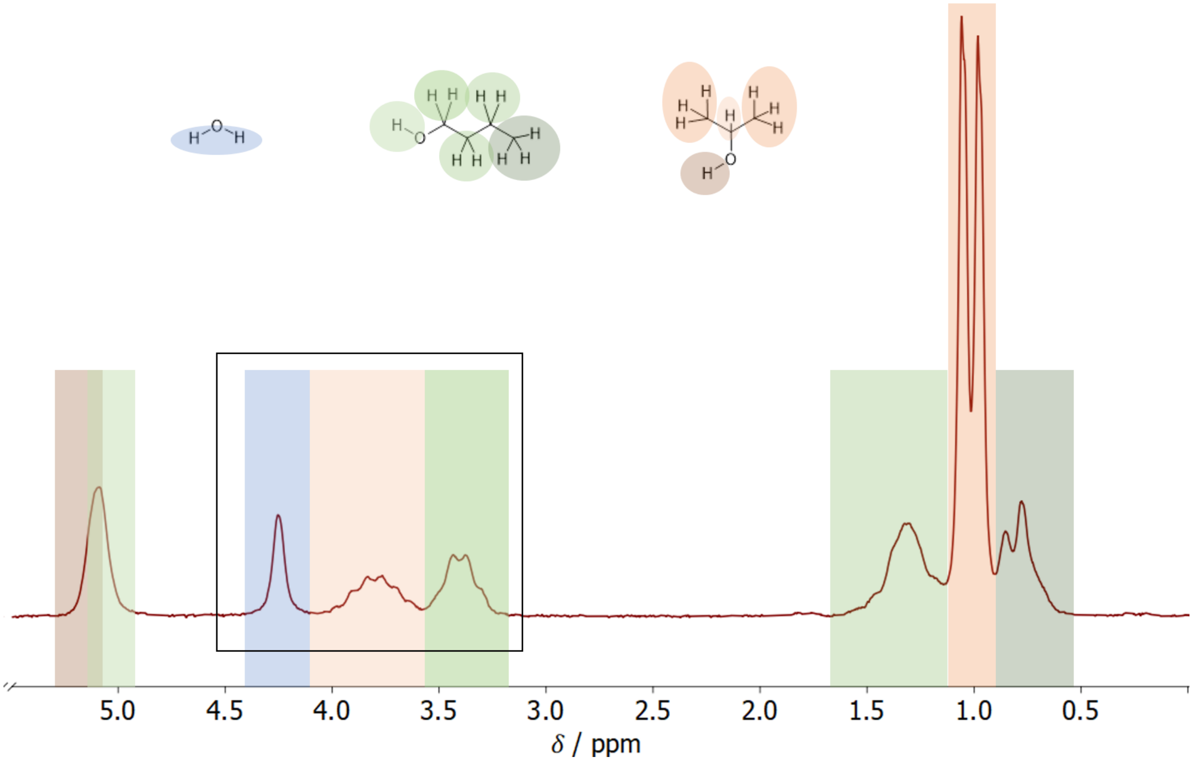

Figure S2: ^{1}H NMR spectrum of a mixture containing (1-butanol + 2-propanol + water). The black box marks the spectral region used for quantitative analysis.

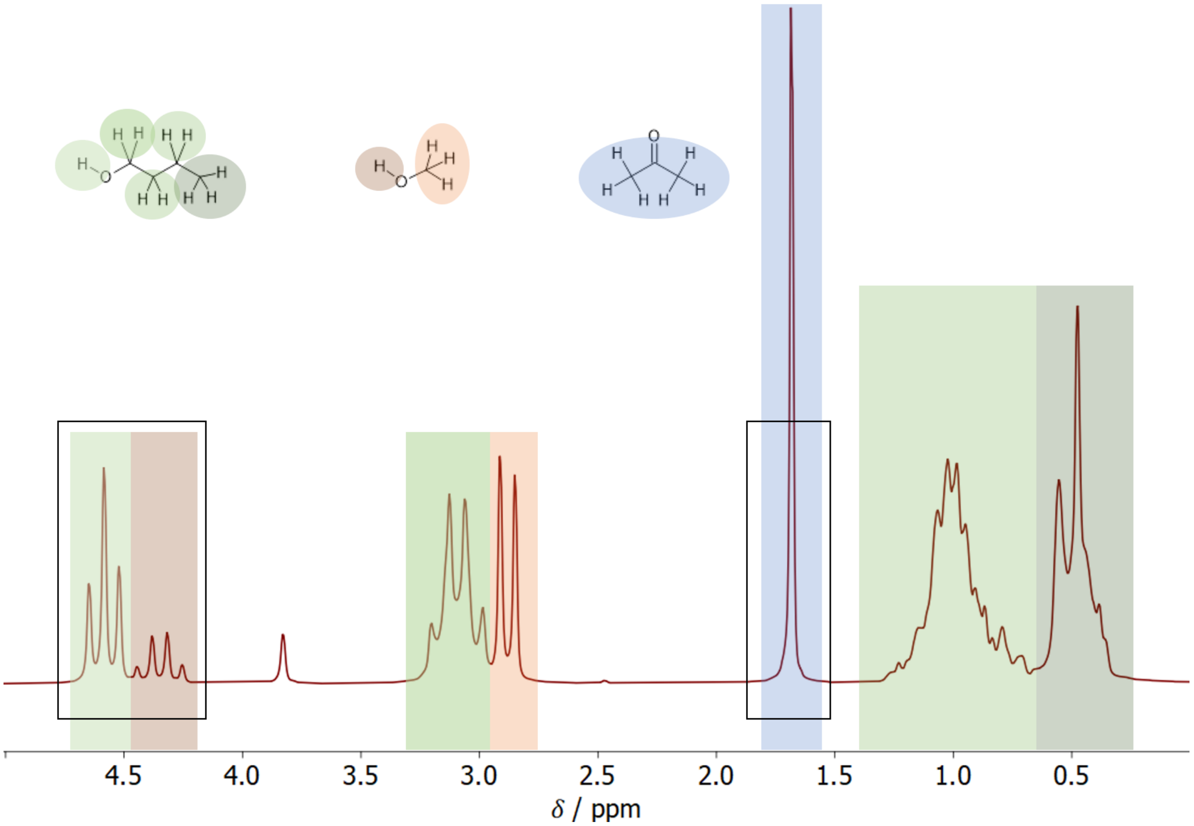

Figure S3: ^{1}H NMR spectrum of a mixture containing (acetone + 1-butanol + methanol). The black box marks the spectral region used for quantitative analysis.

Additional Information on the Mixtures

In Table S3, the chemicals and their manufacturers and purities are given. In the dataset, for each experiment, the analysis certificates of the used chemicals are provided along with the mixture information, if available. In Figures S4 and S5, the residue curves (at 1 bar) of the ternary mixtures used in this work, calculated using extended Raoult's Law with activity coefficients estimated using the NRTL model[8], are given. Both systems (acetone + 1-butanol + methanol) and (1-butanol + 2-propanol + water) show binary azeotropes, as indicated in the figures. Moreover, the system (1-butanol + 2-propanol + water) exhibits a liquid-liquid equilibrium (LLE). Most experiments in the dataset for this system used feed mixtures that avoid process conditions leading to LLE in the plant. Binary interaction parameters τ_{ij} of NRTL were fitted using isobaric experimental binary data at 1 bar from the Dortmund Data

Bank (DDB)[9]. The parameters are given in Table S4.

Table S3: Purities and manufacturers of components used in experimental campaigns in the batch distillation plant. Purity is reported as specified by the supplier's certificates of analysis.

Component	Supplier	Lot-no.	Molar purity
Acetone	Supelco	I1324114	99.9%
1-Butanol	Fisher Thermo Scientific	A0444653	99.9%
Ethanol	Sigma-Aldrich	STBK7309	≥ 99.9%
Methanol	Sigma-Aldrich	STBL4182	99.9%
2-Propanol	Honeywell	M2360	99.99%

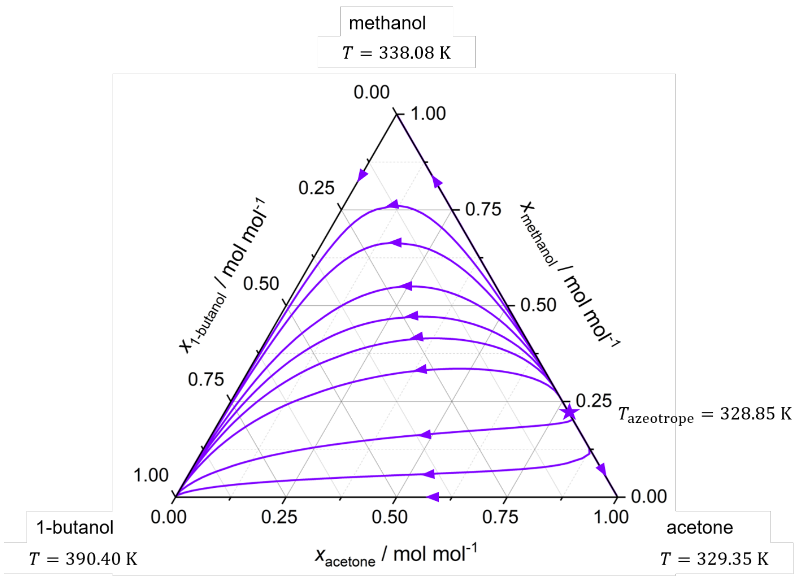

Figure S4: Residue curves for the system (acetone + 1-butanol + methanol) at 1 bar. Azeotropes are indicated with a star symbol. Boiling temperatures of the pure components and the azeotropic mixtures at 1 bar are specified.

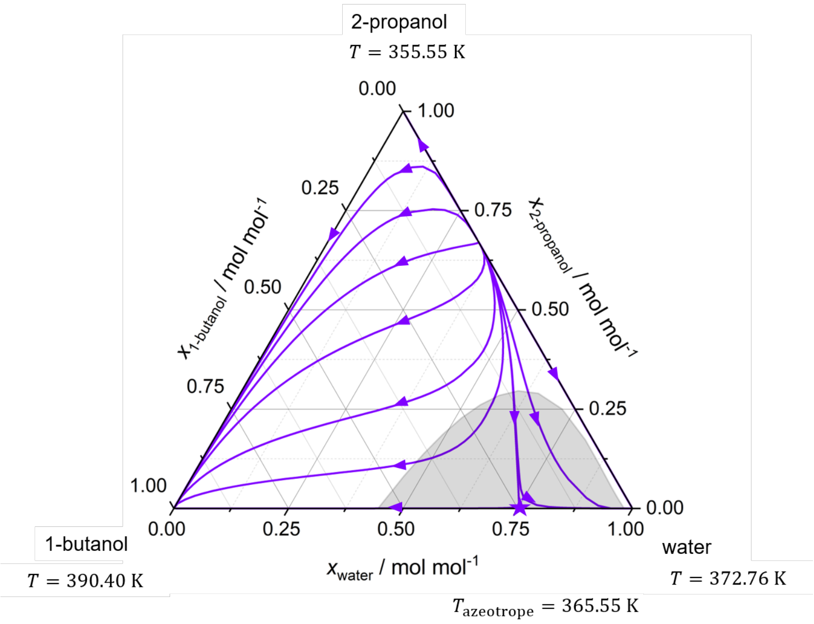

Figure S5: Residue curves for the system (1-butanol + 2-propanol + water) at 1 bar. Azeotropes are indicated with a star symbol. Boiling temperatures of the pure components and the azeotropic mixtures at 1 bar are specified.

Table S4: Interaction parameters τ_{ij} of the NRTL model[8] fitted to experimental data for the binary systems from the DDB[9].

Component i	Component j	τ_{ij}	τ_{ji}
Acetone	1-Butanol	0.69823	-0.06839
Acetone	Methanol	0.29423	0.33341
1-Butanol	2-Propanol	-0.41247	0.46349
1-Butanol	Methanol	0.63230	-0.46038
1-Butanol	Water	0.05864	3.75741
Ethanol	2-Propanol	0.48856	-0.45323
2-Propanol	Water	-0.10456	2.74607

Overview of the Experimental Dataset

In Figure S6, the availability of each data mode is depicted for each experiment in the dataset. Supplementary Table S5 gives an overview over the settings used for each operating point in the dataset alongside number of anomalies observed for each operating point. The order of components in the initial molar mixture composition column is the same as the order of components in the file name.

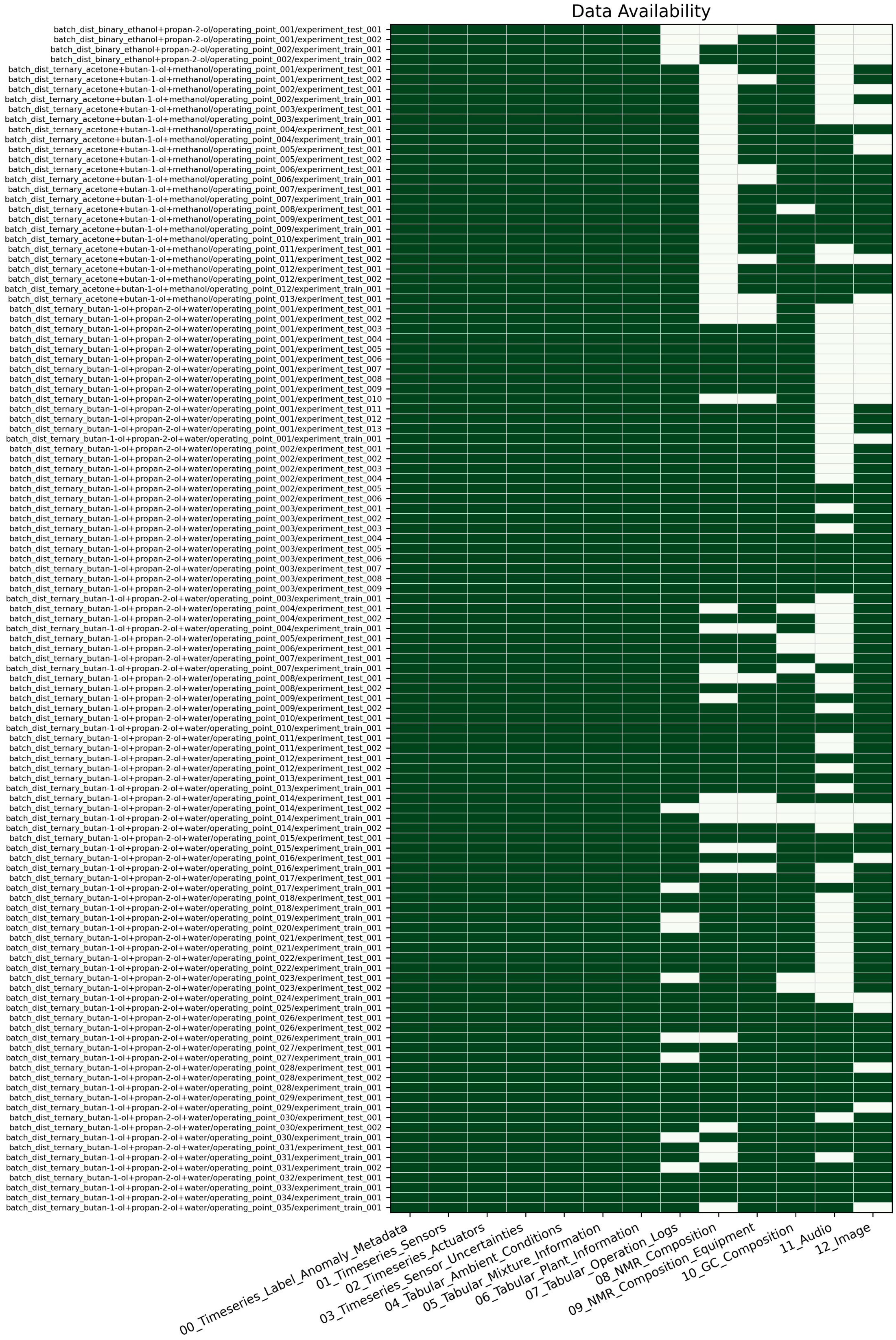

Figure S6: Availability of data modes for each experiment in the dataset. Green tiles mark available data modes, white tiles mark missing data.

References

(1) Brombacher, W. G. *Mercury Barometers and Manometers*; U. S. Department of Commerce, National Bureau of Standards, 1960.

(2) Helmus, J. J.; Jaroniec, C. P. Nmrglue: an open source Python package for the analysis of multidimensional NMR data. *Journal of Biomolecular NMR* **2013**, *55*, 355–367, DOI: 10.1007/s10858-013-9718-x.

(3) Cobas, C. Applications of the Whittaker smoother in NMR spectroscopy. *Magnetic Resonance in Chemistry* **2018**, *56*, 1140–1148, DOI: 10.1002/mrc.4747.

(4) Zhang, F.; Tang, X.; Tong, A.; Wang, B.; Wang, J.; Lv, Y.; Tang, C.; Wang, J. Baseline correction for infrared spectra using adaptive smoothness parameter penalized least squares method. *Spectroscopy Letters* **2020**, *53*, 222–233, DOI: 10.1080/00387010.2020.1730908.

(5) Erb, D. pybaselines: A Python library of algorithms for the baseline correction of experimental data. 2024.

(6) Li, D.-W.; Hansen, A. L.; Yuan, C.; Bruschweiler-Li, L.; Brüschweiler, R. DEEP picker is a deep neural network for accurate deconvolution of complex two-dimensional NMR spectra. *Nature Communications* **2021**, *12*, DOI: 10.1038/s41467-021-25496-5.

(7) Friebel, A.; Specht, T.; von Harbou, E.; Münnemann, K.; Hasse, H. Prediction of flow effects in quantitative NMR measurements. *Journal of Magnetic Resonance* **2020**, *312*, 106683, DOI: 10.1016/j.jmr.2020.106683.

(8) Renon, H.; Prausnitz, J. M. Local Compositions in Thermodynamic Excess Functions for Liquid Mixtures. *AIChE Journal* **1968**, *14*, 135–144, DOI: 10.1002/aic.690140124.

(9) Dortmund Data Bank. 2024; www.ddbst.com.
